# Preserve or Cover? The Isolated Left Vertebral Artery in Totally Endovascular Zone 2 TEVAR

**DOI:** 10.31083/RCM52503

**Published:** 2026-05-25

**Authors:** Antonio Marzano, Luca di Marzo, Wassim Mansour

**Affiliations:** ^1^Vascular Surgery Unit, Department of General and Specialized Surgery and Anesthesiology, “Sapienza” University of Rome, 00161 Rome, Italy

The management of supra-aortic branches during zone 2 thoracic endovascular aortic repair (TEVAR) has significantly evolved over the last decade, driven by the increasing need to achieve a longer and more durable proximal sealing zone, particularly in light of the growing recognition of late distal endograft migration, which may occur even several years after the index procedure [1]. For the left subclavian artery (LSA), the current trend is clear: whenever feasible, preservation or revascularization is increasingly favored, particularly in elective settings and in patients at higher neurologic risk. In contrast, management of an isolated left vertebral artery (ILVA) remains less well defined. Current guidelines recognize the importance of this variant and suggest that vertebral artery preservation should be considered, especially when the left vertebral artery is dominant or collateral reserve is limited [2, 3, 4]. However, robust ILVA-specific evidence remains scarce. A recent systematic review identified only seven retrospective cohort studies (178 patients), and even the most contemporary ILVA-focused endovascular series remain small, with cohorts ranging from 4 to 67 patients, with follow-up from 6 to 63.6 months, and generally low but non-uniform neurologic event rates—from no 30-day neurologic events in the largest multicenter series and no stroke or spinal cord ischemia in smaller fenestration cohorts, to one perioperative ischemic stroke (4.0%) in a 25-patient multicenter Castor (MicroPort Medical, Shanghai, China)-based series [5, 6, 7, 8, 9]. Most recommendations still rely on anatomical findings, retrospective experience, and extrapolation from the broader LSA literature rather than on dedicated comparative datasets [5].

This unresolved issue has become more relevant because zone 2 TEVAR is increasingly performed as a totally endovascular procedure. In fragile, elderly, or comorbid patients, reducing surgical trauma is not simply a technical preference but a strategic necessity [10]. The expansion of endovascular arch technology has made that shift possible. Integrated single-branch devices, second-generation unibody platforms, modular thoracic branch systems, and physician-modified fenestration techniques have all broadened the spectrum of patients who can undergo proximal sealing in zone 2 without surgical debranching [11, 12, 13, 14].

Therefore, the ILVA is no longer an occasional anatomical curiosity: it is a branch whose management may influence both the physiology and the technical architecture of the procedure.

Pu *et al*. [15] should therefore be commended for addressing a question that has long been discussed but not adequately addressed. In their retrospective single-center study of 56 patients with ILVA undergoing zone 2 TEVAR, direct coverage was compared with fenestration-based preservation. The overall technical success rate was 98.21%, there was no perioperative mortality, and major early clinical outcomes were comparable between the groups. The key distinction emerged not in overt perioperative events, but in postoperative vessel alterations. Direct coverage was associated with a significantly greater reduction in ILVA diameter both at discharge and during follow-up, with a numerically higher late occlusion rate, whereas fenestration better maintained vertebral diameter over time [15]. This represents the study’s main contribution. Rather than limiting the discussion to immediate technical feasibility, the authors introduced postoperative vertebral remodeling as a measurable hemodynamic endpoint [15].

Although not a proof of clinical benefit, progressive ILVA narrowing or late occlusion is physiologically relevant because it may reduce vertebrobasilar inflow and posterior-circulation reserve, especially when collateral flow is limited [2, 3, 4, 5, 15, 16].

This is an important observation. The study does not demonstrate that every ILVA must be salvaged, nor does it prove that preserving vessel diameter necessarily translates into lower rates of stroke, spinal cord ischemia, vertebrobasilar insufficiency, or other longer-term neurologic complications. However, it does challenge the longstanding assumption that ILVA coverage is physiologically neutral. If exclusion of the ostium is followed by progressive vertebral narrowing or occlusion in a significant number of patients, then coverage should be understood as a biologically active choice rather than as a benign omission. This is especially relevant in patients with left vertebral dominance, contralateral hypoplasia, incomplete Willisian collateralization, or extensive thoracic aortic coverage, where even modest reductions in posterior circulation reserve may become clinically meaningful [2, 3, 4, 5, 15, 16].

The paper is also valuable because it reopens the technical debate involving totally endovascular zone 2 repair. In the present study, fenestration was achieved with an Ankura thoracic stent-graft (Lifetech Scientific, Shenzhen, China) and was frequently accompanied by bridge stenting of the LSA. This strategy is attractive because it preserves a totally endovascular intervention and avoids a cervical bypass. At the same time, it introduces additional interfaces and branch-dependent variables. By contrast, integrated unibody branch platforms such as Castor, and likely newer second-generation evolutions such as Cratos (MicroPort Medical, Shanghai, China), place the LSA branch into the main graft and may theoretically reduce junctional instability and branch-related leaks compared with bridged or multi-component solutions [17, 18]. Conversely, Castor-based strategies often require upper-extremity surgical exposure because of the decreased caliber when using arm access, whereas standard thoracic graft plus fenestration strategies may allow a more percutaneous workflow.

In practical terms, fenestration-based strategies may be advantageous when procedural flexibility, lower-profile delivery, or a less invasive and potentially more percutaneous workflow is prioritized, whereas integrated unibody branch devices may be preferable when branch alignment is predictable and greater stability with fewer modular interfaces is desired [11, 17, 18]. Thus, the trade-off is not simply preservation versus non-preservation, but rather which preservation strategy offers the best balance among seal, branch durability, access morbidity, and procedural reproducibility.

Ankura-based fenestration strategies also illustrate the practical advantage of procedural adaptability, since the same platform can be combined with different fenestration techniques and adjunctive branch maneuvers according to arch configuration and target-vessel anatomy [7, 9, 15]. By contrast, Castor and newer Cratos-type integrated branch devices offer a more standardized branch design that may be particularly attractive when a short proximal landing zone and branch stability are prioritized, especially in dissection-oriented anatomy, although their application remains influenced by device-specific sizing windows, branch orientation, and upper-extremity access requirements [11, 13, 14]. For that reason, these devices are best viewed not as interchangeable solutions, but as distinct technical options whose applicability depends on pathology, anatomy, operator experience, and local availability.

Another strength of the study is conceptual: it supports an anatomy-first and circulation-aware approach. The ILVA should probably not be treated as an all-or-none problem. There are patients in whom coverage may be tolerated, particularly when the contralateral vertebral artery is robust and the circle of Willis is complete. There are others in whom preservation is more compelling, including patients with dominant left vertebral circulation, concomitant need for extensive aortic coverage, prior infrarenal or thoracoabdominal aortic repair, the presence of a patent left internal mammary graft in Coronary Artery Bypass Grafting (CABG) patients, or any anatomy suggesting reduced collateral flow. Preservation becomes more compelling when preoperative imaging suggests that ILVA exclusion would materially reduce posterior circulation or spinal cord collateral reserve, such as in left vertebral dominance, contralateral vertebral hypoplasia or occlusion, incomplete circle of Willis or poor posterior communicating arteries, extensive planned thoracic coverage, or previous infrarenal or thoracoabdominal aortic repair [2, 3, 4, 15, 16]. The challenge is that this decision-making process remains underinvestigated. Current reports, including the recent systematic review on ILVA management, remain limited by small numbers, heterogeneity of the pathology, mixed open, hybrid, and endovascular cohorts, and non-uniform neurologic endpoints [5, 6, 7, 8, 9, 15, 19].

The limitations of the present analysis are therefore as important as its message. This was a retrospective study from a single high-volume center; the sample size was small because ILVA is an uncommon variant; the fenestration group was older; the duration of computed tomography angiography (CTA) follow-up differed between the groups; and discrete neurologic endpoints were not the primary focus. Most importantly, no systematic brain magnetic resonance imaging (MRI), perfusion computed tomography (CT), transcranial Doppler, or formal assessment of circle-of-Willis hemodynamics was performed [15].

These limitations may have influenced both the magnitude and the interpretation of the observed differences: the small sample size reduces statistical power for hard clinical endpoints, the older age of the fenestration group may have introduced differences in baseline risk factors, and non-uniform CTA follow-up may have affected the apparent extent of vertebral remodeling or late occlusion over time [15].

In view of these limitations, the next generation of studies should move beyond technical patency alone. The field now needs multicenter registries and collaborative comparative studies that incorporate vertebral dominance, intracranial collateral anatomy, silent cerebral ischemia on diffusion-weighted MRI, standardized assessment of posterior-circulation stroke and spinal cord ischemia, serial vessel patency/remodeling on imaging, and patient-centered neurologic and functional outcomes. Only then will we know whether maintaining ILVA patency is simply elegant or genuinely protective.

Pu *et al*. [15] have not closed the debate; they have improved it. Their work suggests that ILVA salvage during zone 2 TEVAR is more than a technical issue, but may have measurable hemodynamic value. At present, the most rational position is a selective one: preserve the ILVA whenever posterior circulation reserve appears vulnerable and is essential to avoid ischemic complications, and whenever that preservation can be achieved without compromising procedural safety or seal quality.

A pragmatic clinical approach may therefore be to first determine whether the ILVA will be directly jeopardized by the intended proximal seal and to assess its functional importance by vertebral dominance, contralateral vertebral patency and caliber, and intracranial collateral anatomy preoperatively. A second step is to identify factors that may further reduce posterior circulation or spinal cord reserve, including extensive planned thoracic coverage, prior infrarenal or thoracoabdominal aortic repair, or associated LSA exclusion. If collateral reserve appears robust, direct coverage may be reasonable; if reserve is uncertain or clearly limited, preservation should be favored. When preservation is indicated, the choice should then shift from whether to preserve to how to preserve, selecting the technique that best maintains seal quality and procedural safety in the specific anatomy, whether by fenestration-based reconstruction or by an integrated single-branch platform [2, 3, 4, 5, 11, 15, 16].

Until more comparative data become available, this is the most defensible course based on current evidence and contemporary endovascular practice.

## Abbreviations

TEVAR, thoracic endovascular aortic repair; LSA, left subclavian artery; ILVA, isolated left vertebral artery; CTA, computed tomography angiography; CT, computed tomography; MRI, magnetic resonance imaging.
